# The Extent of Human Apolipoprotein A-I Lipidation Strongly Affects the β-Amyloid Efflux Across the Blood-Brain Barrier *in vitro*

**DOI:** 10.3389/fnins.2019.00419

**Published:** 2019-05-16

**Authors:** Roberta Dal Magro, Sara Simonelli, Alysia Cox, Beatrice Formicola, Roberta Corti, Valeria Cassina, Luca Nardo, Francesco Mantegazza, Domenico Salerno, Gianvito Grasso, Marco Agostino Deriu, Andrea Danani, Laura Calabresi, Francesca Re

**Affiliations:** ^1^School of Medicine and Surgery, Nanomedicine Center NANOMIB, University of Milano-Bicocca, Monza, Italy; ^2^Dipartimento di Scienze Farmacologiche e Biomolecolari, Centro Grossi Paoletti, Università degli Studi di Milano, Milan, Italy; ^3^Istituto Dalle Molle di Studi sull’Intelligenza Artificiale, Scuola Universitaria Professionale della Svizzera Italiana, Università della Svizzera Italiana, Manno, Switzerland

**Keywords:** HDL, apoA-I, β-amyloid, Alzheimer’s disease, blood-brain barrier

## Abstract

Much evidence suggests a protective role of high-density lipoprotein (HDL) and its major apolipoprotein apoA-I, in Alzheimer’s disease (AD). The biogenesis of nascent HDL derived from a first lipidation of apoA-I, which is synthesized by the liver and intestine but not in the brain, in a process mediated by ABCA1. The maturation of nascent HDL in mature spherical HDL is due to a subsequent lipidation step, LCAT-mediated cholesterol esterification, and the change of apoA-I conformation. Therefore, different subclasses of apoA-I-HDL simultaneously exist in the blood circulation. Here, we investigated if and how the lipidation state affects the ability of apoA-I-HDL to target and modulate the cerebral β-amyloid (Aβ) content from the periphery, that is thus far unclear. In particular, different subclasses of HDL, each with different apoA-I lipidation state, were purified from human plasma and their ability to cross the blood-brain barrier (BBB), to interact with Aβ aggregates, and to affect Aβ efflux across the BBB was assessed *in vitro* using a transwell system. The results showed that discoidal HDL displayed a superior capability to promote Aβ efflux *in vitro* (9 × 10^-5^ cm/min), when compared to apoA-I in other lipidation states. In particular, no effect on Aβ efflux was detected when apoA-I was in mature spherical HDL, suggesting that apoA-I conformation, and lipidation could play a role in Aβ clearance from the brain. Finally, when apoA-I folded its structure in discoidal HDL, rather than in spherical ones, it was able to cross the BBB *in vitro* and strongly destabilize the conformation of Aβ fibrils by decreasing the order of the fibril structure (-24%) and the β-sheet content (-14%). These data suggest that the extent of apoA-I lipidation, and consequently its conformation, may represent crucial features that could exert their protective role in AD pathogenesis.

## Introduction

Apolipoprotein A-I (apoA-I) is involved in the generation and metabolism of high-density lipoproteins (HDL) (Zannis et al., 2006). The 28 kDa protein is synthesized in the small intestine and liver, and lipidated with cholesterol and phospholipids by the membrane-bound ATP-binding cassette transporter A1 (ABCA1) to create different subclasses of plasma HDL particles, including discoidal ones. Subsequently, a second lipidation step, mediated by LCAT, is required for maturation of nascent HDL into mature spherical lipid-rich HDL (Oram and Heinecke, 2005; Curtiss et al., 2006). During the HDL maturation process the secondary structure of apoA-I slightly changes (Sevugan Chetty et al., 2012). Therefore, in humans, HDL consist of heterogeneous subclasses, characterized according to charge, size, density, protein, and/or lipid composition. HDL are involved in removing excess cholesterol from peripheral tissues, e.g., the arterial wall, and transporting it to the liver for secretion by reverse cholesterol transport. HDL exert other protective effects, including anti-oxidative, anti-inflammatory, anti-apoptotic, and anti-infective actions (Kingwell et al., 2014).

It is well established that plasma levels of HDL cholesterol (HDL-c) and apoA-I, the major protein component of plasma HDL, inversely correlate with the development of many disorders, including cardiovascular diseases, diabetes, obesity, cancer, and infectious diseases. Dysregulated HDL metabolism has also been linked to brain disorders: a decrease in plasma levels of HDL-c and/or apoA-I are risk factors for memory decline and neurodegenerative diseases, such as Alzheimer’s disease (AD) (Vitali et al., 2014).

Alzheimer’s disease is the most common type of dementia and affects tens of millions of people worldwide. The amyloid hypothesis (Haass and Selkoe, 1993; Glenner and Wong, 2012; Selkoe and Hardy, 2016) proposes that β-amyloid peptide (Aβ), the main component of senile plaques, is the key player in AD pathogenesis. Aβ monomers, derived from the proteolytic cleavage of the larger glycoprotein amyloid precursor protein, if not efficiently cleared from the brain can aggregate into different assemblies, which can then form regular fibrils and plaques.

Some evidence suggests that apoA-I might be involved in the pathogenesis of AD, but its role has not yet been elucidated. In a recent publication, Robert et al. (2017a) suggest that the risk to develop AD could be reduced when supported by high concentrations of plasma levels of HDL and apoA-I. It has also been shown that higher plasma levels of HDL and apoA-I are directly correlated with an increased MMSE and a lower risk of developing AD (Merched et al., 2000). Moreover, a reduction of hippocampal and whole brain volume and of cortical thickness has been detected in AD patients, along with lower plasma levels of apoA-I (Hye et al., 2014). In another study, reduced serum apoA-I has been detected in AD, contrarily to non-demented age-matched patients (Shih et al., 2014). This body of evidence suggests that apoA-I could play a protective role in AD pathogenesis, possibly by promoting the clearance of Abeta from the brain. In favor of this hypothesis are also *in vivo* studies on AD mouse animal models, showing that cognitive deficit is prevented by apoA-I overexpression (Lewis et al., 2010), while it is worsened by its deletion (Lefterov et al., 2010), and i.v., administration of HDL reduces soluble Aβ brain levels (Robert et al., 2016).

However, more research is needed to clarify its implications and no information is available concerning the possible role of distinct HDL subclasses. In this study, we investigate *in vitro* the ability of apoA-I in different lipidation states to affect Aβ efflux across the BBB, using a transwell system made by immortalized human brain capillary endothelial cells.

Moreover, it is known that apoA-I is present in the brain (Elliott et al., 2010), but its mRNA has not been detected (Mahley et al., 1984; Harr et al., 1996; Huang et al., 2008). Thus, brain apoA-I is believed to be plasma derived from peripheral HDL that cross the blood-brain barrier (BBB) (Roheim et al., 1979; Pitas et al., 1987; Stukas et al., 2014; Fung et al., 2017).

Therefore, within the present study we have also investigated how the extent of apoA-I lipidation (giving rise to different HDL subclasses) affects the traversing of the BBB and the interaction with Aβ aggregates; an issue that, to the best of our knowledge, has never been investigated before.

## Materials and Methods

### Human Samples

Human plasma samples from healthy donors were provided by the Immunohematology and Transfusion Medicine Service (SIMT) of ASST Grande Ospedale Metropolitano Niguarda, Milan. All experimental protocols were approved by license 446-092014 CE from Ospedale Niguarda Ca’ Granda and carried out in accordance with these guidelines and regulations. Informed consent was obtained from all donors. Plasma was prepared by low speed centrifugation at 4°C and lipoprotein isolation started within 6 h of blood collection.

### Purification of apoA-I-HDL From Human Plasma

High-density lipoprotein (*d* = 1.063–1.21 g/mL) were purified from human plasma of healthy blood donors by sequential ultracentrifugation. Purified lipoproteins were dialyzed against saline immediately before use and were representative of the total HDL pool of human plasma. This sample was treated with chymase, which degrades small discoidal HDL particles, to obtain a fraction containing only spherical apoA-I-HDL (Favari et al., 2004). Briefly, apoA-I-HDL plasma pool was incubated with granule remnants isolated from rat mast cells (30°μg/ml of granule remnant total protein, equal to 40 BTEE Units/ml) for 2 h at 37°C. After incubation, tubes were placed on ice and centrifuged at 4°C, 12,000 rpm, for 5 min to remove the granule remnant-bound chymase, and the chymase-free supernatants were collected. The lack of discoidal, apoA-I-containing HDL was verified by non-denaturing two-dimensional (2-D) electrophoresis, in which agarose gel electrophoresis was followed by non-denaturing gradient gel electrophoresis (GGE), and immunoblotting against apoA-I. Spherical apoA-I-HDL were characterized by AFM, as described below.

### Preparation of Discoidal HDL

Apolipoprotein A-I was purified from human plasma by gel-filtration chromatography (Bernini et al., 1996) and its purity (>95%) was confirmed by SDS-polyacrylamide gel electrophoresis (SDS-PAGE) using Coomassie protein staining, as previously described (Schägger and von Jagow, 1987). This sample represents lipid-poor apoA-I, having few bound phospholipid molecules. Discoidal apoA-I-HDL were prepared by the cholate dialysis method using palmitoyl-oleoyl-phosphatidylcholine (POPC) and apoA-I in the weight ratio of 2.5:1 (Calabresi et al., 1997). Their size was estimated by non-denaturing GGE using precast 4–30% gels and the Pharmacia Phast System (Franceschini et al., 2013). Phospholipid content of lipid-poor apoA-I and discoidal HDL was measured by an enzymatic method and apoA-I concentration was measured by Lowry method. The final preparation of discoidal HDL contained 2 apoA-I and 205 POPC molecules. Discoidal HDL were characterized by AFM, as described below.

### Atomic Force Microscopy Imaging of apoA-I-HDL

Atomic force microscopy (AFM) imaging was performed using a Nanowizard II (JPK Instruments, Berlin) scanning probe microscope operating in tapping and contact mode in air. In tapping mode imaging of Aβ_1-42_ fibrils, RTESP-300 (Bruker, United States) cantilevers were used with a nominal force constant of 40 N/m, a resonance frequency of 300 kHz, and a nominal tip radius 8 nm. For contact mode imaging of HDL subtypes, DNPS-10 (Bruker, United States) cantilevers were used with a nominal force constant of 0.06 N/m, and a nominal tip radius 10 nm. A detailed protocol used for the apoA-I-HDL characterization is supplied as Supplementary Information (see section “Characterization of HDL by AFM Imaging”). A wide range of areas of AFM images were analyzed using the commercial JPK image processing software and a customized image-analysis software (Matlab, MathWorks Inc, United States).

### Preparation and Characterization of Aβ Aggregates

Aβ_1-42_ (Sigma–Aldrich, Milan, Italy) fibrils were prepared as described (Gregori et al., 2010; Bana et al., 2014; Mancini et al., 2016). Briefly, the peptide (1 mg/mL) was solubilized in 1,1,3,3,3-hexafluoro-2-propanol (HFIP; Sigma–Aldrich, Milan, Italy), dried, resuspended in DMSO at a concentration of 5 mM, and bath sonicated for 10 min. To obtain a fibril-enriched preparation, samples were diluted to 200μM in 10 mM HCl and incubated at 37°C for 72 h. Aβ fibrils were characterized by AFM (Gregori et al., 2010). For the fibrillation process, samples were diluted to 100μM in 10 mM HCl and incubated at 37°C. In the reported images, Aβ was diluted to a final concentration of 10°μM in 10 mM HCl and deposited on freshly cleaved mica. Images were acquired in air in tapping mode.

### Preparation and Characterization of the *in vitro* Model of Blood-Brain Barrier

The *in vitro* BBB model was prepared and characterized as previously described (Mancini et al., 2016), using immortalized human brain endothelial cells (hCMEC/D3 cells) from Institut National de la Sante et de la Recherche Medicale, Paris, France. Briefly, hCMEC/D3 were seeded (60,000 cells/cm^2^) onto collagen-coated (8°μg/cm^2^ rat tail collagen type 1; Gibco, Thermo Fisher Scientific) transwell filters (polycarbonate 12-well, pore size 0.4°μm, translucent membrane inserts 1.12 cm^2^; Costar) to establish a polarized monolayer. The cell monolayer separates two compartments, an apical one (0.5 ml) representing the blood and a basolateral one (1 ml) representing the brain. Cells were grown for 3 days in complete EBM-2 medium (1.4°μM hydrocortisone, 10 mM HEPES, 1% penicillin-streptomycin, 5°μg.mL^-1^ ascorbic acid, and 1% chemically defined lipid concentrate) supplemented with 1 ng.mL^-1^ basic fibroblast growth factor and 10% fetal bovine serum (FBS). After 3 days *in vitro* (DIV), medium was changed to complete EBM-2 medium supplemented with 5% FBS, and 10 mM LiCl and grown for a further 3 days. *Trans*-endothelial electrical resistance (TEER) was monitored with STX2 electrode Epithelial Volt-Ohm meter (World Precision Instruments, Sarasota, FL, United States). The formation of junctions was evaluated by confocal microscopy (LSM710, Carl Zeiss, Oberkochen, Germany) and by measuring the endothelial permeability (EP) to [^14^C-sucrose] (0.5°μCi) and [^3^H]-propranolol (0.5°μCi), as described (Bana et al., 2014). Cell viability was assessed by MTT assay (Bana et al., 2014).

### Effect of apoA-I Lipidation on *in vitro* Aβ Efflux Across the BBB

Five hundred nanomolar of Aβ fibrils dissolved in 1 ml of complete culture medium were placed in the basolateral compartment of the transwell system, as previously described (Mancini et al., 2016). The impact of this treatment on the cell monolayer was evaluated by monitoring TEER, EP to radiolabeled probes, and cell viability, following the procedure described above. 5 nmol/ml of apoA-I in discoidal HDL, spherical HDL, or total HDL plasma pool (Merino-Zamorano et al., 2016) dissolved in 500°μl of PBS was added to the apical compartment of the transwell. The impact on hCMEC/D3 monolayers of the treatments was determined by measuring the TEER after 3–24 h of incubation with apoA-I-HDL in the apical side of transwells. After different incubation times (up to 3 h) at 37°C, aliquots from the apical compartment were collected and the Aβ content was measured by ELISA assay (IBL International, Italy). The Aβ EP across the cell monolayer from the basolateral to the apical compartment (defined as Aβ efflux) was calculated as described (Bana et al., 2014; Mancini et al., 2016). As controls, 5 nmol/ml of commercially available apoA-I(Sigma-Aldrich, Milano, Italy) was dissolved in PBS and added to the apical compartment. The Aβ efflux was determined as previously described.

### Effect of Lipidation of apoA-I on Its Ability to Cross the BBB *in vitro*

A total of 5 nmol/ml of apoA-I in discoidal HDL, spherical HDL, or total HDL plasma pool dissolved in 500°μl of PBS was added to the apical compartment of the transwell and incubated at 37°C. After different times (up to 3 h) of incubation, the amount of apoA-I in the basolateral compartment was measured by ELISA assay (IBL International, Italy), and EP was calculated as described (Bana et al., 2014; Mancini et al., 2016).

### Effect of apoA-I Lipidation on Preformed Aβ Fibrils

Fifty micromolar Aβ_1-42_ fibrils were incubated at 37°C in buffer B (PBS 15 mM and NaCl 20 mM, pH 7.4) with discoidal HDL, spherical HDL, or apoA-I-HDL plasma pool, containing 0.18 nmol/ml of apoA-I in order to mimic its low brain concentration (Roheim et al., 1979; Fagan et al., 2000). After different times of incubation an aliquot from each sample was immobilized on a freshly cleaved mica substrate, rinsed with Milli-Q water, dried under a gentle stream of nitrogen, and analyzed by AFM.

The effect of apoA-I lipidation on preformed Aβ fibrils was also monitored by ThT assay (Quan et al., 2016). Briefly, 2°μM of Aβ fibrils were added with 5°μM ThT (Sigma-Aldrich, Milan, Italy), 10 mM glycine buffer pH 8.5 in 1 cm^2^ × 1 cm^2^ fluorimeter quartz cuvettes equipped with hermetic tips to prevent evaporation. 0.0036 nmol/ml of apoA-I in discoidal HDL, spherical HDL, or total HDL plasma pool were added to the sample and the ThT fluorescence (ex. 450 nm; em. 485 nm) was monitored at 37°C under stirring with a FP8500 fluorimeter (Jasco) equipped with a 4-cells peltier-thermostated sample holder. Low Aβ and apoA-I concentrations (25-fold less than in the AFM experiments) were utilized for ThT experiments since fibrils produce sizeable scattering of the fluorescence excitation light, and in order to maintain the Aβ:apoA-I molar ratio as in the AFM experiments.

### Molecular Modeling of apoA-I

The recently obtained discoidal HDL with apoA-I (Bibow et al., 2017) without helix-5 was considered the starting point for this work (PDB ID: 2N5E). It was demonstrated by chemical shift comparison and lipid paramagnetic relaxation enhancement experiments (Bibow et al., 2017) that apoA-I with and without helix 5 are structurally similar, strongly indicating that the overall structure of apoaA-I is preserved in the shortened construct considered in the present study. One hundred 1,2-dimyristoyl-sn-glycero-3-phosphocholine (DMPC) lipid molecules were inserted as previously described (Bibow et al., 2017). This model was solvated and neutralized by adding 0.15 M Na and Cl ions. CHARMM36 force field (Huang and MacKerell, 2013) was used to define protein and lipids topologies, and TIP3P model (Jorgensen et al., 1983) was used for the water molecules. The obtained system was minimized by applying 1,000 steps of steepest descent energy minimization algorithm, followed by preliminary NVT of 200 ps. V-rescale thermostat was applied to maintain the temperature at 300 K with a time constant of 0.1 ps (Bussi et al., 2007). An NPT of 200 ps was carried out at 300 K (τ = 1 ps) and 1 atm (τ = 5 ps). V-rescale (Bussi et al., 2007) and Berendsen (Berendsen et al., 1984) coupling methods were used as temperature and pressure coupling. Finally, molecular dynamics (MD) equilibration of 100 ns was performed to optimize the DMPC/apoA-I complex.

### Computational Docking and MD of apoA-I in Complex With Aβ Fibril

To determine the initial orientation of Aβ fibrils on DMPC/apoA-I complex, a pentamer of Aβ_17-42_ was extracted from the PDB model 2BEG (Lührs et al., 2005), and considered for docking experiments (Yu and Zheng, 2012). In this NMR model, each peptide monomer has the disordered N-terminal residues 1–16 missing. However, the remaining residues 17–42 were suggested to contribute the stability of the mature fibril mostly and were included in the simulation models here, as described in previous computational studies (Fan et al., 2015; Grasso et al., 2018). The Aβ_17-42_ model was first docked on apoA-I using Patchdock (Schneidman-Duhovny et al., 2005). The top-scored 100 conformations were subjected to Firedock (Andrusier et al., 2007; Mashiach et al., 2008) to refine and rescore docking solutions. The top ranked molecular system was solvated in a cubic box of 13 nm^3^× 11 nm^3^× 8 nm^3^ and neutralized by counterions. Each system consisted of approximately 120,000 interacting particles. CHARMM36 force field (Huang and MacKerell, 2013) was used to define protein and lipids topologies, while TIP3P model (Jorgensen et al., 1983) was used for water molecules. The system was first minimized by applying the steepest descent energy minimization algorithm. Three different replicas of the same system were generated with different initial velocities to increase the statistics of MD data. A preliminary MD simulation of 100 ps was performed in NPT ensemble at 300 K (τ = 1 ps) and 1 atm (τ = 5 ps) by applying position restraints on the heavy atoms of the solute. V-rescale (Bussi et al., 2007) and Berendsen (Berendsen et al., 1984) coupling methods were used as temperature and pressure coupling. Finally, three production simulations were performed at 300K for 100 ns. For comparison, Aβ_17-42_ alone in water was also simulated. Principal component analysis (PCA) was applied to reduce the dimensionality of the system (Deriu et al., 2016a; Grasso et al., 2016), elucidating large-scale and low-frequency modes, thus yielding collective motions related to the destabilization of the Aβ_17-42_ fibril (Maisuradze et al., 2009). After the alignment of Aβ_17-42_ C-α Cartesian coordinates, the covariance matrix was calculated and diagonalized. To estimate the structural order of the Aβ_17-42_ model and to what extent fibrils chains are aligned, an order parameter was calculated for each MD snapshot using equation (1):

$$(1)ordP=\frac{1}{N_{r}}\overset{42}{\underset{r=17}{\Sigma }}\frac{\langle v_{r},z\rangle }{\left||v_{r}||\cdot ||z|\right|}=\frac{1}{N_{r}}\overset{42}{\underset{r=17}{\Sigma }}\cos\alpha$$

where *N*, is the number of residues along the peptide chain; v_r_ is the vector joining each of the Cα-atoms pertaining to chain A with the corresponding Cα-atom (same residue number) of chain E; and z is the fibril axis. Values of ordP close to 1 indicate the amyloid-like shape alignment, whereas values of ordP < 1 are typical of a distorted structure.

### Statistical Analysis

Data are expressed as mean ± SEM or SD obtained from three independent experiments, each of them in triplicate. Data were analyzed with one-tailed Student’s *t* test for MD analysis. Data from *in vitro* transwell assays were analyzed with one-way ANOVA and the Tukey’s *post hoc* test was applied. *p*-value < 0.05 was considered significant.

## Results

### Characterization of apoA-I-HDL

**FIGURE 1:**
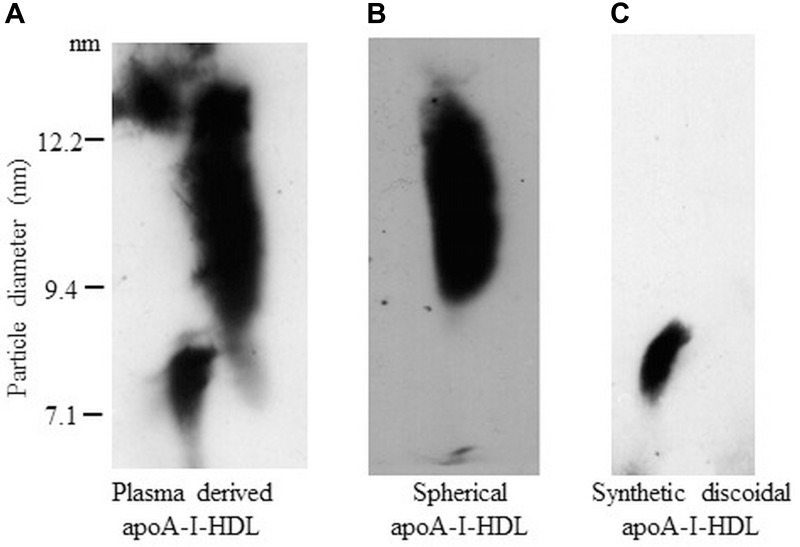
Characterization of HDL by 2D-GGE. Total HDL, purified from human plasma, were separated by two-dimensional electrophoresis and transferred onto a nitrocellulose membrane, on which different HDL subclasses were detected by immunoblotting using anti-human apoA-I antibody. **(A)** Plasma derived apoA-I-HDL (total plasma HDL). **(B)** Spherical apoA-I-HDL, derived from plasma HDL preparation after incubation for 2 h at 37°C with chymase-containing granule remnants, which degrades small discoidal HDL particles. **(C)** Synthetic discoidal apoA-I-HDL, prepared by the cholate dialysis method.

Plasma derived HDL were analyzed by 2D-GGE (Figure 1A). Left smear represents the subclass of discoidal apoA-I-HDL and right smear represents the subclass of spherical apoA-I-HDL, as previously described (Didichenko et al., 2016). About 10–14% of apoA-I-HDL plasma pool is represented by discoidal apoA-I-HDL. This sample was treated with chymase in order to selectively degrade discoidal apoA-I-HDL, obtaining a preparation containing mature spherical apoA-I-HDL. In Figure 1B, the complete degradation of discoidal particles is evident, indicated by the disappearance of their smear. Synthetic discoidal apoA-I-HDL were also characterized by 2D-GGE (Figure 1C). As expected, the discoidal particles migrate in the pre-beta region. The estimated diameter of HDL by 2D-GGE is 11 ± 2 nm for spherical HDL and 8.8 ± 0.2 nm for synthetic discoidal HDL.

**FIGURE 2:**
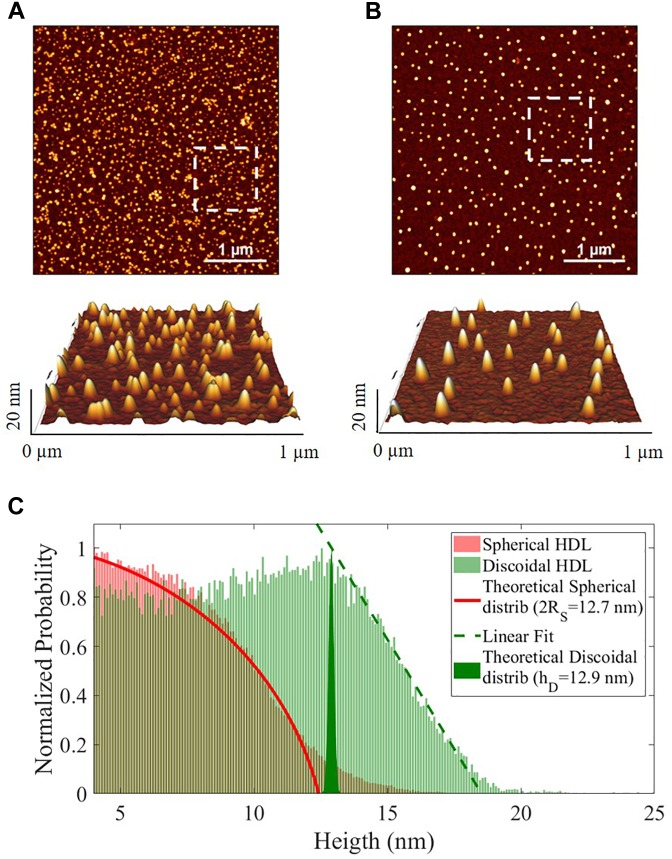
Representative images of the morphology of HDL subclasses obtained by AFM imaging. **(A)** Discoidal and **(B)** spherical HDL on APTES functionalized mica (4 μm^2^ × 4 μm^2^, 1024 × 1024-pixel, Z-scale 20 nm), with 3-dimensional projections of a 1 μm^2^ × 1 μm^2^ region (white squares in the images). **(C)** Normalized height statistical distributions of spherical (red) and discoidal (green) HDL in the range 4–25 nm. The red fit is the theoretical distribution for a sphere (2R_S_ = 12.7 nm), the green filled area represents the δ-function (hD = 12.9 nm) and the green dotted line is the linear regression for the final part of the distribution.

Spherical and discoidal apoA-I-HDL were also characterized by AFM imaging (Supplementary Figure 2). Both HDL subclasses displayed visual homogeneity in size distribution and morphology (Figure 2A,B). The height statistical distributions of AFM imaging allowed discrimination between the spherical and discoidal shapes (Figure 2C) and showed that the average dimensions of the two HDL subclasses were comparable (spherical apoA-I-HDL diameter 2R_S_ 12.7 nm, discoidal apoA-I-HDL size 12.9 nm). It should be noted that the HDL sizes obtained by using 2D-GGE and AFM techniques agree qualitatively, even if there are inherent technical differences between the two methods, which do not allow direct comparison of the results.

The extent of apoA-I lipidation, determined by measuring the phospholipid content, was 92:1 mmol lipids/mmol apoA-I for synthetic discoidal apoA-I and 15:1 mmol lipids/mmol apoA-I for lipid-poor apoA-I, as calculated from our own and previously published data (Jayaraman et al., 2012).

### Characterization of Aβ Fibrillation

**FIGURE 3:**
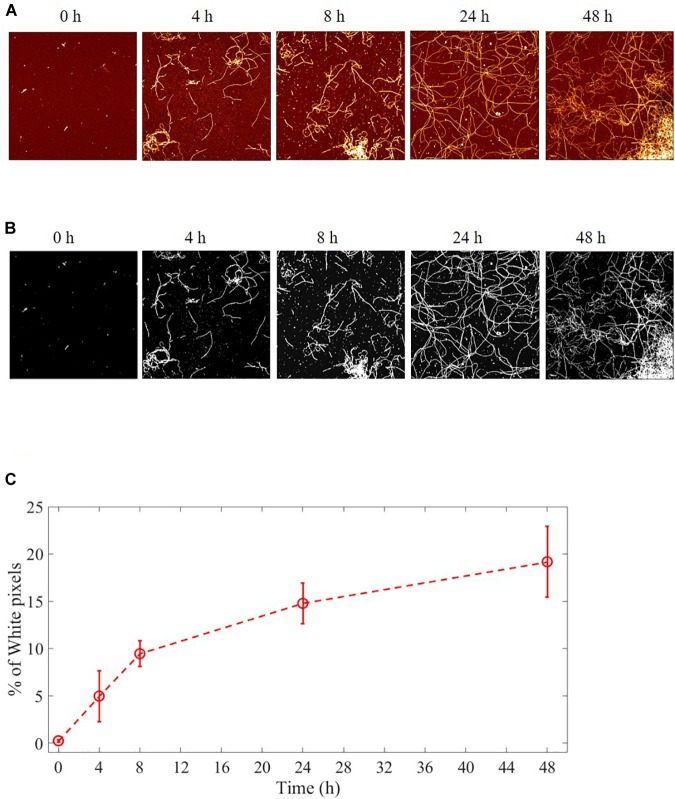
Aggregation process of Aβ from monomers to fibrils studied by AFM. Representative images of Aβ at different fibrillation stages (*t* = 0, 4, 8, 24, and 48 h) of incubation at 37°C. **(A)** AFM images: 4 μm^2^ × 4 μm^2^, 1024 × 1024-pixel, Z-scale 10 nm. **(B)** A fixed height threshold (here 1.5 nm) is applied to the AFM images in order to quantify the number of pixels above this threshold, expressed in terms of percentage with respect the total number of pixels. The number of such pixels is proportional to the total length of the fibril i.e., to the sum of the lengths of all the deposited fibrils. In this way, it is possible to quantify the fibril growth. **(C)** Quantification of the fibrillation. Percentage of pixel above a height threshold (1.5 nm) as obtained from AFM images, plotted as a function of the incubation time at 37°C.

The aggregation process of Aβ from monomers to fibrils was followed and characterized by AFM imaging. Several images were acquired at the beginning of the fibrillation (*t* = 0h) and at successive fixed times (*t* = 4, 8, 24, and 48 h). Representative images are illustrated in Figure 3A. The results showed that long fibrils were formed over time, as previously observed (Gregori et al., 2010). The aggregation is characterized by progressive formation of unbranched fibrils of constant diameter and increasing length.

The process of fibril growth can be quantitatively evaluated by considering the time evolution of the number of pixels above a fixed height threshold in the AFM images of fibril morphology (Supplementary Figures 1, 2). Figure 3B shows AFM images of Aβ fibrils at different incubation times with a height threshold of 1.5 nm. The percentage of pixels above a certain threshold increases with fibril extension and density, and it can be considered as a quantitative index of the aggregation process. Threshold pixel percentages over time (Figure 3C) show that there is continuous Aβ fibril growth in length up to 48 h.

### Characterization of *in vitro* BBB Model

**FIGURE 4:**
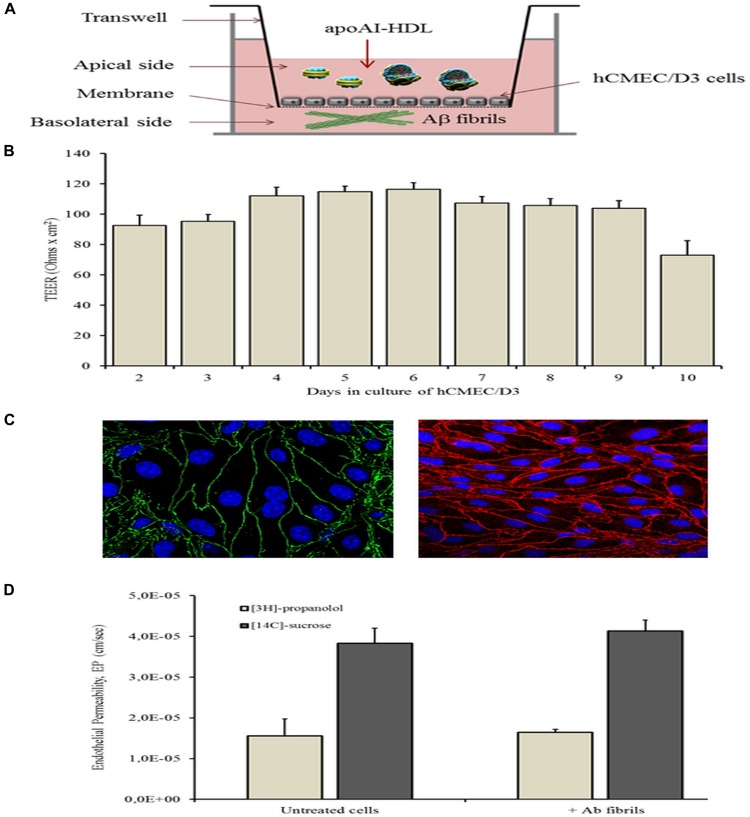
*In vitro* BBB model. The *in vitro* BBB model, made in a transwell system by culturing hCMEC/D3 cells was characterized by monitoring the TEER values over time, by probing the formation of tight junctions and by measuring the para- and transcellular passage of radioactively labeled probes. **(A)** Graphical representation of BBB model. **(B)** TEER values monitored from 2 to 10 days in culture. **(C)** confocal microscopy visualization of tight and adherens junctions (blue, nuclei; green, ZO-1; and red, VE-cadherin). **(D)** endothelial permeability of [^14^C]-sucrose, as probe for paracellular permeability, and [^3^H]-propranolol, as probe for transcellular permeability, before and after incubation with Aβ fibrils in the basolateral compartment of the transwell system.

The *in vitro* BBB model was prepared and characterized (Figure 4). The TEER was monitored over time and the results showed that the maximum value (116.37 ± 4.37 Ω/cm^2^) was registered 6 days after seeding (Figure 4B). At this time point, the formation of junctions was checked by confocal microscopy and by measuring the paracellular and transcellular EP of radiolabeled sucrose and propranolol, respectively. The results showed that Claudin-5 (Figure 4C, stained in green) and VE-Cadherin (Figure 4C, stained in red), two key components of tight and adherens endothelial junctions, are formed in the hCMEC/D3 monolayer 6 days after seeding. The EP of [^3^H]-propranolol and [^14^C]-sucrose was 1.56 ± 0.13 × 10^-5^ and 3.83 ± 0.84 × 10^-5^ cm/min, respectively, suggesting that tight junctions had formed (Figure 4D). At 6 days after seeding, 500 nM of Aβ fibrils (characterized by AFM images, see Figure 3) were added to the basolateral compartment of the transwell system and the impact of fibrils on cell monolayer properties was checked. After 3h of hCMEC/D3 monolayer exposure to fibrils, TEER did not significantly change (114.10 ± 4.82 Ω cm^2^) nor did EP of *trans*- and para-cellular probes (Figure 4D). Moreover, the treatment did not affect cell viability (>95% cells viability with respect to untreated cells), as assessed by MTT assay.

The impact on hCMEC/D3 monolayers of the different subclasses of apoA-I-HDL was determined by measuring the TEER after 3 and 24 h of incubation with particles in the apical side of transwells. The results showed that the treatments of cell monolayers with HDL did not affect their bioelectrical properties, with TEER values of 114 ± 11 Ω⋅cm^2^ after treatment (Supplementary Table 1).

### Effect of apoA-I Lipidation on *in vitro* Aβ Efflux Across the BBB

The Aβ efflux from the basolateral compartment of the transwell when apoA-I in different lipidation states were present in the apical compartment was measured by ELISA assay (Supplementary Figure 5).

**FIGURE 5:**
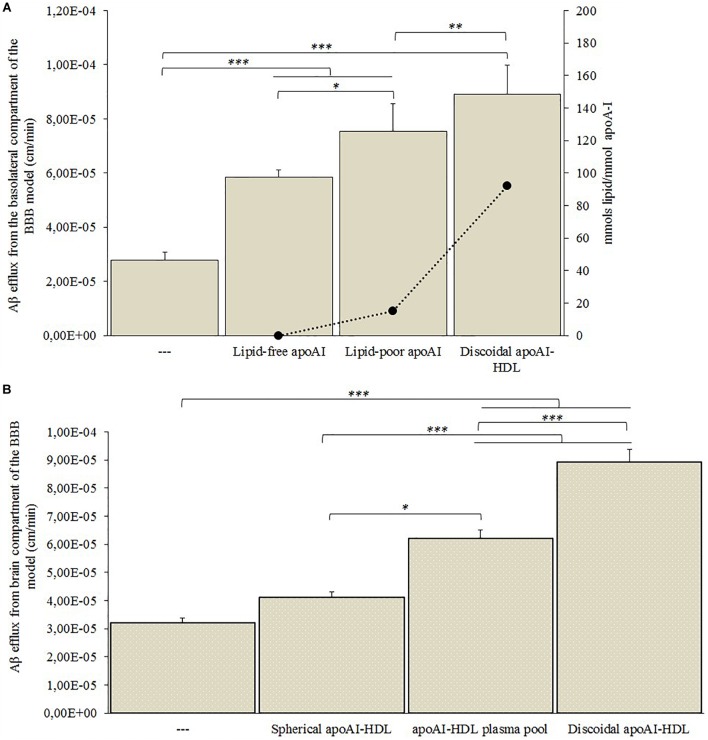
Aβ efflux across the BBB model in the presence of human plasma-derived HDL. hCMEC/D3 cells were cultured on a transwell system and 500 nM of Aβ fibrils was added to the medium in the basolateral compartment. Different subclasses of HDL or Aβ-binding proteins were added to the apical compartment and the Aβ efflux from the basolateral compartment was measured by ELISA assay. **(A)** Endothelial permeability (EP) values of Aβ in the presence of lipid-free, lipid-poor or discoidal apoA-I, and the ratio of lipids to apoA-I in each sample. PBS alone in the apical compartment was used as a control (—). **(B)** EP values of Aβ in the presence of spherical apoA-I-HDL, apoA-I-HDL plasma pool or discoidal apoA-I-HDL. PBS alone in the apical compartment was used as a control (—). Data were expressed as endothelial permeability (EP), reported as the mean ± SEM of triplicate experiments. One-way ANOVA found significant differences **(A)** *F*_(3,32)_ = 87.47; *p* = 0.00001; **(B)** *F*_(3,32)_ = 120.61; *p* = 0.00001. ^∗^*p* < 0.05, ^∗∗^*p* < 0.01, ^∗∗∗^*p* < 0.01 by Tukey’s *post hoc* test.

The presence of apoA-I in the apical compartment significantly increased Aβ efflux from the basolateral side of the BBB model (Figure 5A). In particular, the ability of apoA-I to enhance the Aβ efflux increased with the increase of its lipidation state, reaching maximum Aβ efflux when discoidal apoA-I-HDL were present in the apical compartment.

The discoidal apoA-I-HDL significantly increased Aβ efflux from the basolateral side of the BBB model, compared to the apoA-I-HDL plasma pool (*p* = 0.0016) and spherical apoA-I-HDL (*p* = 0.011) (Figure 5B).

### *In vitro* BBB Crossing of apoA-I-HDL

**FIGURE 6:**
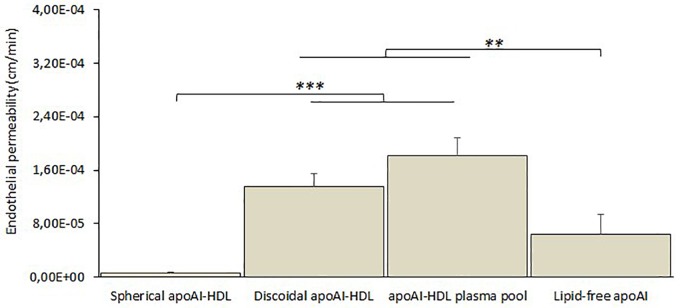
*In vitro* BBB crossing of different subclasses of human plasma HDL. The passage of apoA-I in different lipidation states (spherical apoA-I-HDL, discoidal apoA-I-HDL, apoA-I-HDL plasma pool, or lipid-free apoA-I) across the hCMEC/D3 monolayer was determined by measuring apoA-I in the lower compartment of the transwell system at different incubation times (up to 3 h) by ELISA assay. Data were expressed as endothelial permeability (EP), reported as the mean ± SEM of triplicate experiments. One-way ANOVA found a significant difference [*F*_(3,32)_ = 75.14; *p* = 0.00001]. ^∗∗^*p* < 0.01, ^∗∗∗^*p* < 0.01 by Tukey’s *post hoc* test.

Different subclasses of apoA-I-HDL were added to the apical compartment of the transwell system and their EP across the cell monolayer was estimated by measuring the apoA-I content in the basolateral compartment by ELISA assay for up to 3 h. The results (Figure 6) showed that discoidal HDL displayed higher EP values (with a passage of 1.8 ± 0.6% of apical apoA-I), compared to spherical ones (*p* = 0.004), and lipid-free apoA-I (*p* = 0.048).

### Effect of apoA-I Lipidation on Preformed Aβ Fibrils

**FIGURE 7:**
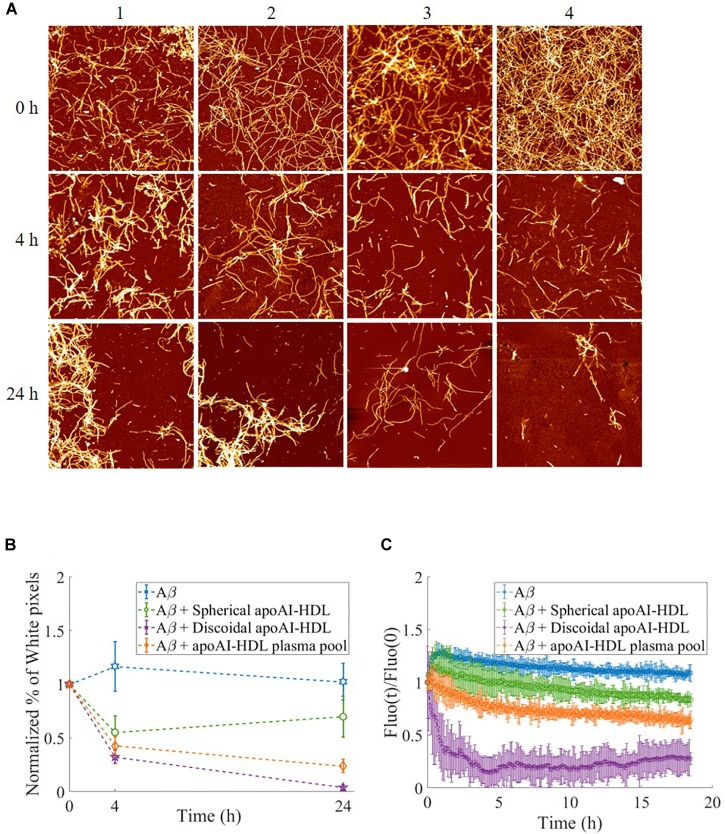
Disaggregation of preformed Aβ fibrils in the presence of HDL. **(A)** Representative AFM images of Aβ fibrils over time, incubated at 37°C either alone (column 1) or with different HDL subclasses: spherical HDL (column 2), total HDL plasma pool (column 3), discoidal HDL (column 4). Images are 4 μm^2^ × 4 μm^2^, 1024 × 1024-pixel, Z-scale 10 nm. **(B)** The normalized percentage of pixels with a height above a threshold of 1.5 nm (white pixel percentage) is reported for Aβ in the presence of the different HDL subclasses at different incubation times. Values are the average of pixels higher than the threshold over several images acquired on the same sample. Error bars represent SD Each sample is normalized to its respective starting point (value at *t* = 0 h). **(C)** Thioflavine T fluorescence as a function of time in samples containing 2 μM Aβ fibrils alone (blue dotted) or incubated with spherical HDL (green dotted), total HDL pool (orange dotted), or discoidal HDL (purple dotted). The intensities were normalized to the respective zero-time intensity.

The effect of apoA-I lipidation on the disaggregation of preformed Aβ fibrils was assessed by AFM and thioflavine T (ThT) assay (Quan et al., 2016). Aβ fibrils were incubated with apoA-I in different lipidation states for up to 24 h and changes in the morphology of fibrils were followed by AFM imaging (Figure 7). The results showed that, starting from mature Aβ fibrils of comparable length (Figure 7A, row 1), the incubation with spherical apoA-I HDL did not induce significant changes in fibril morphology, and concentration (Figure 7A, column 2) compared to Aβ fibrils alone (Figure 7A, column 1). On the contrary, incubation with both the apoA-I-HDL plasma pool (Figure 7, column 3) and discoidal apoA-I-HDL (Figure 7A, column 4) induced a strong time-dependent reduction of fibril concentration and extension.

The percentage of pixels above the 1.5 nm threshold (determined as reported in Supplementary Figure 1) normalized with respect to the starting point (value at *t* = 0 h) is reported for each sample at different times to obtain a quantitative analysis of AFM images. The results (Figure 7B) demonstrate the superior capability (1.6-fold increase) of discoidal apoA-I-HDL in disassembling preformed Aβ fibrils compared to spherical apoA-I-HDL.

The β-sheet content of Aβ fibril samples were analyzed using a ThT fluorescence assay and the results (Figure 7C) showed that when fibrils are in incubation alone or with spherical apoA-I-HDL, their β-sheet content did not significantly change. On the contrary, the presence of apoA-I-HDL plasma pool induced a 40% reduction in β-sheet content after 18 h, and there is an almost complete disruption of β-sheet structures within 4 h in the presence of discoidal apoA-I-HDL.

### Discoidal apoA-I-HDL Induce a Structural Destabilization of Aβ

**FIGURE 8:**
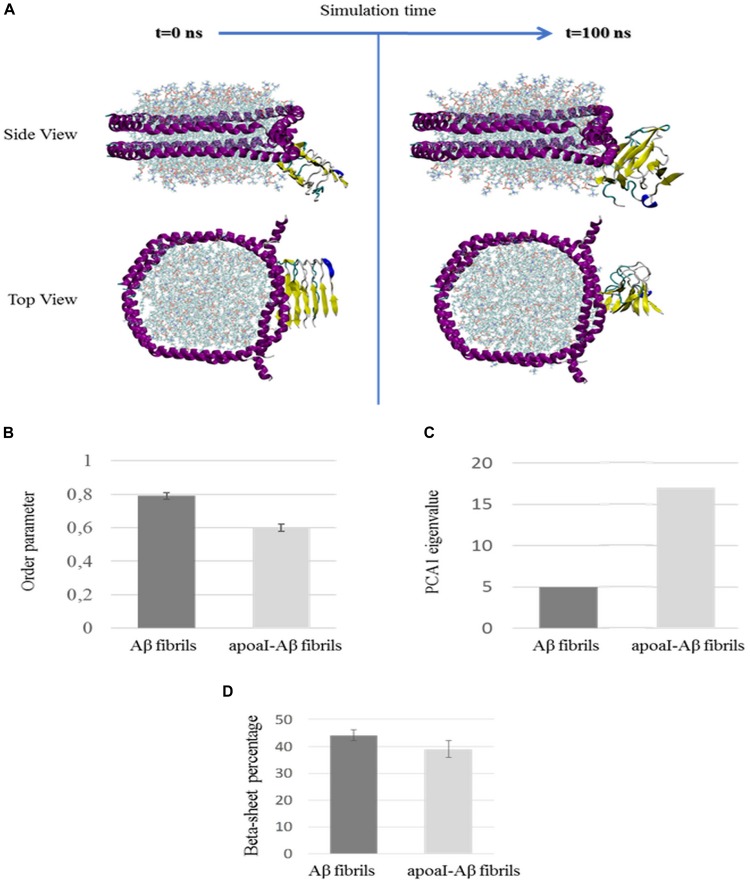
Molecular dynamic simulation analysis. **(A)** Visual inspection of the apoA-I interaction with Aβ fibril at the beginning (*t* = 0 ns) and at the end (*t* = 100 ns) of the MD simulation. The side-view and top-view representations are reported in the upper and lower panels, respectively. Yellow represents Aβ_17-42_ and magenta represents apoA-I. **(B–D)** The destabilization of Aβ fibril by discoidal apoA-I-HDL obtained by MD simulations is shown. **(B)** the order parameter calculated at the equilibrium, **(C)** the eigenvalue of the first PCA vector, and **(D)** the β-sheet content of Aβ fibril alone in water and in complex with apoA-I.

The interaction between discoidal apoA-I-HDL and Aβ_17-42_ was evaluated using MD simulations. Protein structural stability was analyzed by monitoring the time evolution of the root mean square deviation (RMSD) of Aβ_17-42_ in water and Aβ_17-42_ in complex with apoA-I. Three different replicas of the Aβ_17-42_ alone in water and in complex with apoA-I were examined to increase the statistics of the MD data. It was observed that protein conformational stability was reasonably reached in the last 20 ns of the simulations (Supplementary Figure 3). The apoA-I-Aβ_17-42_ contact surface, characterized by protein-lipid (Aβ-1,2-dimyristoyl-sn-glycero-3-phosphocholine) and protein-protein (Aβ-apoA-I) interactions, covers 6.3 ± 2 nm^2^ of solvent accessible surface. The hydrophobic interaction plays a pivotal role in the contact area (Supplementary Figure 4). The visual inspection of the apoA-I-Aβ_17-42_ complex through the MD simulation is reported in Figure 8A and highlights the conformational destabilization of the Aβ_17-42_ due to the interaction with apoA-I.

The previously highlighted conformational instability can be quantified by analyzing the fibril order parameter (*ordP*), as reported in Figure 8B. A significantly decreased *ordP* value (ordP < 1 are typical of a distorted structure) was found in the case of apoA-I-Aβ_17-42_ (*ordP* = 0.60 ± 0.02) compared to Aβ_17-42_ alone in water (*ordP* = 0.79 ± 0.02). The PCA provides another image, which highlights the large-scale and low frequency modes mainly related to the distortion of the Aβ fibril. After the alignment of the Aβ C-α atoms, the covariance matrix was calculated, and diagonalized for each simulated system (Aβ_17-42_ in water and Aβ_17-42_ in complex with apoA-I). The amplitude of the first Principal Component Vector, which takes into account more than 50% of the total variance of the protein motion, is reported in Figure 8C, highlighting a marked increase of conformational fluctuations when Aβ_17-42_ is in complex with apoA-I (eigval_PCA1_ = 17) compared to Aβ_17-42_ alone in water (eigval_PCA1_ = 5). Finally, a decreased β-sheet content was observed by computing the secondary structures probabilities (Figure 8D) along the Aβ fibril chains at the equilibrium, as previously described (Deriu et al., 2014, 2016b; Grasso et al., 2015, 2016, 2017; Janaszewska et al., 2017). Despite the beta-sheet percentage decreases only by 14%, this is a statistically significant molecular event observed only in the apoAI-Aβ molecular system in the simulated time-scale of 100 ns. Moreover, the marked loss of structural order, quantified by fibril order parameter, will further affect the beta-sheet percentage in longer time-scale. Within this framework, our results clearly show a molecular event not observed in the Aβ system in water environment, i.e., the ability of apoA-I protein to affect the conformational stability of Aβ in the investigated time-scale of 100 ns.

We note that, notwithstanding the different experimental and simulative approaches, the ThT and simulation techniques both suggest a relevant role in Aβ destabilization induced by discoidal HDL.

## Discussion

Considerable evidence suggests that plasma HDL, as well as having vasoprotective functions, could exert a protective role in AD (Kingwell et al., 2014; Vitali et al., 2014), and but the mechanisms involved have not been thoroughly investigated. Since Aβ clearance from the brain, a way to counteract the onset or progression of AD, partially occurs across the brain vasculature (Bates et al., 2009), and it is essential to understand if and how circulating HDL might affect Aβ passage across the BBB.

Considering previously published data about the ability of HDL and apoA-I to bind Aβ *in vitro* (Koldamova et al., 2001; Paula-Lima et al., 2009; Shih et al., 2014) and reduce Aβ levels in the brain of AD animal models (Robert et al., 2016), we hypothesized that plasma-derived HDL acts by accelerating the Aβ egress from the brain to the blood via “sink effect,” as already speculated for different Aβ binding molecules or particles (Golabek et al., 1995; Mancini et al., 2016).

To investigate this issue, we used a simple BBB model consisting of an hCMEC/D3 monolayer that separates a basolateral compartment containing Aβ fibrils to mimic the AD brain from an apical one containing different human HDL subclasses, and mimicking the blood. We chose to carry out the experiments on fibrils, since we have already shown, using the same BBB model system utilized in the present investigation, that oligomers are able to exit from the brain side either spontaneously or by “sink effect” induced by Aβ-binding liposomes (Mancini et al., 2016).

The results showed that the presence of apoA-I in the apical compartment of the transwell system strongly enhanced the Aβ egress from the basolateral one. This effect is dependent on the lipidation of apoA-I, reaching the maximum Aβ efflux when apoA-I is folded in discoidal HDL. Actually, no effect on Aβ efflux was detected when apoA-I was contained in mature spherical HDL. It should be noted that other lipid-based nanoparticles functionalized with Aβ ligands (Mancini et al., 2016) showed the ability to promote Aβ clearance, however with a lower efficiency compared to discoidal HDL.

Our results suggest the possibility that the apoA-I conformation, which depends on its lipidation state, may be also involved. In fact, the flexible apoA-I molecule adapts its structural motif to stabilize the different HDL subclasses(Silva et al., 2008; Phillips, 2013).

Therefore, we theorize that the plasma profile of HDL subclasses could differ between healthy and AD patients, thus affecting Aβ clearance from the brain. A recent study showed that HDL from AD patients were less functional compared to HDL from healthy subjects (Camponova et al., 2017), supporting this idea. Since only small soluble Aβ assemblies, and not fibrils, are able to cross the endothelial monolayer (Mancini et al., 2016), and since apoA-I is not synthetized in the brain (Demeester et al., 2000; Koch et al., 2001), the herein shown ability of apoA-I-HDL to promote the Aβ clearance across the BBB should assume their ability to cross the barrier and to disaggregate fibrils.

To verify this speculation, we tested the ability of HDL subclasses to cross the BBB *in vitro*.

Our results show that when apoA-I folded its structure in discoidal HDL, rather than in spherical particles, it was able to cross the BBB *in vitro*, suggesting that the lipidation state of apoA-I could be a key determinant affecting these features. Moreover, we found that the apical/basolateral ratio of apoA-I in our model system is comparable with the plasma/CSF ratio. Concerning the mechanism of apoA-I crossing, it should be noted that previous work suggests that the SR-BI receptor is highly selective for lipid uptake, and excludes apoAI and apoAII (Acton et al., 1996; Gillard et al., 2017). Therefore, our speculation is that apoA-I crosses the BBB through a mechanism involving the LDL receptor-related protein family, as previously suggested (Merino-Zamorano et al., 2016), and that the shape and size of HDL could be additional determining factors (Fernández-de-Retana et al., 2017).

To the best of our knowledge, the data about the ability of different HDL subclasses to cross the BBB reported in this investigation have never been published before.

Finally, we investigated the effect of different apoA-I-HDL subclasses on pre-formed Aβ fibrils by AFM imaging, ThT assay, and MD simulation. AFM imaging showed that the presence of discoidal apoA-I-HDL strongly reduced the amount and concentration of long fibrils. This was confirmed by ThT assay, where a strong, and rapid reduction of the β-sheet content of fibrils was detected. The molecular modeling results highlighted the conformational destabilization of Aβ upon its interaction with apoA-I when associated to discoidal HDL. A significant distortion of the fibril order and a decrease in the β-sheet content was identified, clearly suggesting a key role played by discoidal apoA-I-HDL in destabilizing the Aβ fibrils.

Taken all together the data reported in present investigation, suggest that apoA-I-HDL action is not only peripheral via “sink effect,” but also central after BBB crossing.

In summary, we can speculate that at earliest stages of AD, discoidal apoA-I-HDL species in plasma, more than alternatively lipidated HDL species, may be involved in synergic activity with brain discoidal apoA-I-HDL pool: the central HDL pool maintains Aβ in a soluble form, while the peripheral HDL pool enhances its efflux from the brain. These results add new information to previously published knowledge (Robert et al., 2016, 2017b; Slot et al., 2017), suggesting that the lipidation state of apoA-I, and its conformation may be an important determinant for its role in preventing β-amyloidosis, and therefore may influence the pathogenesis of AD.

Our results are also in agreement with a previously published study about the ability of apoA-I-HDL to promote clearance of Aβ through the cerebral vessel (Robert et al., 2017b) in a more advanced 3D cerebrovascular model. Our data adds new insights about the potential ability of discoidal HDL to cross the BBB and to destroy Aβ aggregates enhancing its clearance from the brain.

Considering that the main brain apoprotein, apoE, promotes Aβ aggregation (Dafnis et al., 2016), we can speculate that the ratio with brain apoA-I, promoting disaggregation, may be an important key point in modulating the peptide aggregation/disaggregation paradigm. Moreover, considering that apoE in the blood is able to promote Aβ clearance from the brain (Robert et al., 2017b) its coupling with the disaggregating activity of brain apoA-I could play an important role in brain Aβ clearance. Finally, considering that Aβ cleared into the blood interacts with circulating apoA-I, preventing its aggregation and reducing its accumulation in the vasculature (Handattu et al., 2009; Lefterov et al., 2010; Lewis et al., 2010; Robert et al., 2016, 2017a; Fernández-de-Retana et al., 2017) depicts a complex interplay among these apolipoproteins in the brain and in the blood.

However, the synergic action between different lipoproteins in modulating Aβ aggregation/disaggregation and transport across the BBB will be an important issue to be investigated.

As a final consideration, it should be pointed out that the results herein reported have been obtained using an *in vitro* model. Evidently, the neurovascular unit (NVU), the area where these processes naturally occur, is considerably more complex than endothelial cells alone (Sagare et al., 2012). Indeed, the brain capillary endothelial cells of the BBB do not function alone, but they work in synergy within a context of a multicellular NVU, which includes neurons, astrocytes, pericytes, and microglia and the blood vessels themselves (McConnell et al., 2017). The NVU components cooperate to regulate cerebrovascular function and permeability through the vasculature, which has unique structure along the vascular tree in the brain (Andreone et al., 2017). Thus, different structures can contribute to Aβ exchange across the BBB. Accordingly, our results should be taken as an indication that will deserve confirmation on a model closer to the physiological state (Robert et al., 2017a) or *in vivo* in animal models.

## Ethics Statement

Human plasma samples from healthy donors were provided by the Immunohematology and Transfusion Medicine Service (SIMT) of ASST Grande Ospedale Metropolitano Niguarda, Milan, Italy. All experimental protocols were approved by license 446-092014 CE from Ospedale Niguarda Ca’ Granda and carried out in accordance with these guidelines and regulations. Written informed consent was obtained from each donor and all donors were over the age of 18.

## Author Contributions

RD performed *in vitro* experiments on the blood-brain barrier model. SS purified, prepared, and characterized the different subtypes of HDL from human plasma. AC performed *in vitro* experiments with beta-amyloid peptide. BF prepared and characterized beta-amyloid aggregates. RC and VC performed AFM imaging experiments. LN performed ThT fluorescence assays. DS performed statistical analysis of data. GG, MD, and AD performed the molecular modeling analysis. LC, FM, and FR contributed to the data interpretation and participated in the drafting of the manuscript. FR coordinated the study, designed the experiments, and analyzed the data. All authors contributed to the manuscript revision, read and approved the submitted version, and agreed to be accountable for all the aspects of the work.

## Conflict of Interest Statement

The authors declare that the research was conducted in the absence of any commercial or financial relationships that could be construed as a potential conflict of interest.
