# Supplementary material for: The Extent of Human Apolipoprotein A-I Lipidation Strongly Affects the β-Amyloid Efflux Across the Blood-Brain Barrier in vitro
Source: Front Neurosci. 2019 May 16;13:419. doi: 10.3389/fnins.2019.00419 (PMC6532439; doi:10.3389/fnins.2019.00419)
Supplement: Supplementary file 1 [file Table_1.docx]

**Supplementary Material**

**The Extent Of Human Apolipoprotein A-I Lipidation Strongly Affects The β-Amyloid Efflux Across The Blood-Brain Barrier *in vitro***

**Roberta Dal Magro^1‡^, Sara Simonelli^2‡^, Alysia Cox^1*^, Beatrice Formicola^1^, Roberta Corti^1^, Valeria Cassina^1^, Luca Nardo^1^, Francesco Mantegazza^1^, Gianvito Grasso^3^, Marco Agostino Deriu^3^, Andrea Danani^3^, Laura Calabresi^2^ and Francesca Re^1^**

^1^ School of Medicine and Surgery, Nanomedicine Center NANOMIB, University of Milano-Bicocca, Monza, Italy.

^2^ Centro Grossi Paoletti, Dipartimento di Scienze Farmacologiche e Biomolecolari, Università degli Studi di Milano, Milano, Italy.

^3^ Istituto Dalle Molle di Studi sull'Intelligenza Artificiale (IDSIA), Scuola Universitaria Professionale della Svizzera Italiana (SUPSI), Università della Svizzera Italiana (USI), Manno, Switzerland.

^‡^ These Authors contributed equally to this work.

*** Correspondence:**

Alysia Cox

a.cox1@campus.unimib.it

**Keywords: HDL, apoA-I, β-amyloid, Alzheimer’s disease, blood-brain barrier**

1. **Supplementary Data and Figures**

**1.1 Characterization of HDL by AFM imaging**

AFM measurements allow the discrimination of the different geometrical appearances of HDL subtypes through determination of their morphological characteristics. Considering the intrinsic tip convolution (finite size and specific geometry of the AFM cantilever tip) and the relatively small dimensions of the samples, the aspect of discoidal and spherical HDL cannot be clearly distinguished by AFM imaging. However, these image limitations can be overcome by considering the shape of the height statistical distributions presented in Fig. 2. Indeed, as result of AFM imaging procedure it is possible to associate to each (x, y) coordinate of the image, the quantity z which represents the measured height at the (x, y) coordinate. By analyzing the statistical distribution of height, for discoidal HDL (in green), the distribution of the height values ranging between 4 nm and 25 nm is significantly different from the height distribution of spherical HDL (in red). Here, only the heights > 4 nm are studied in order to eliminate the substrate contributions (height too close to the mica surface i.e. z≈0). In order to quantify the resulting statistical outcomes, we consider the theoretical height distribution for a sphere and fit the normalized spherical HDL height histograms (Fig. S1, in red) with the formula theoretically predicted for a sphere:

$P(h)=n_{0}\left( 2\pi\sqrt{{R_{s}}^{2}-{({h-R}_{s})}^{2}} \right)$.

Where $n_{0}$ is the normalization factor and $R_{s}$ is the mean radius of the sphere. We obtained a ${2R}_{s}$=12.7 nm with a coefficient of determination $R^{2}$=0.96.

For discoidal HDL (Fig. S1, green histogram) the theoretical height distribution can be considered a δ-function centered on the disk’s average height $h_{D}$. The height distribution is influenced by the tip convolution, the polydispersity of the sample and the possible different deposition orientation of the disks. Since the data cannot be fit with the δ-function, the mean disks height is considered the maximum height of the distribution. To simplify the visualization of the maxima, the linear regression of the final part of the distribution is shown (Fig. 2). By this procedure, the average height value for discoidal HDL was 12.9 nm. We can confirm the superior capability of height distribution statistical analysis in discriminating between different HDL subtypes, which exhibit comparable radial projections.

**1.2 From images to numbers: quantification by using a height threshold**

By applying a threshold to every AFM image, it was possible to measure the number of pixels whose height was above this threshold (white pixels). The number of white pixels was directly proportional to the total quantity of Aβ aggregated in fibrils in the field of view. Supplementary Fig. 1 represents the Fig. 7A in the manuscript after the application of the threshold (1.5 nm).


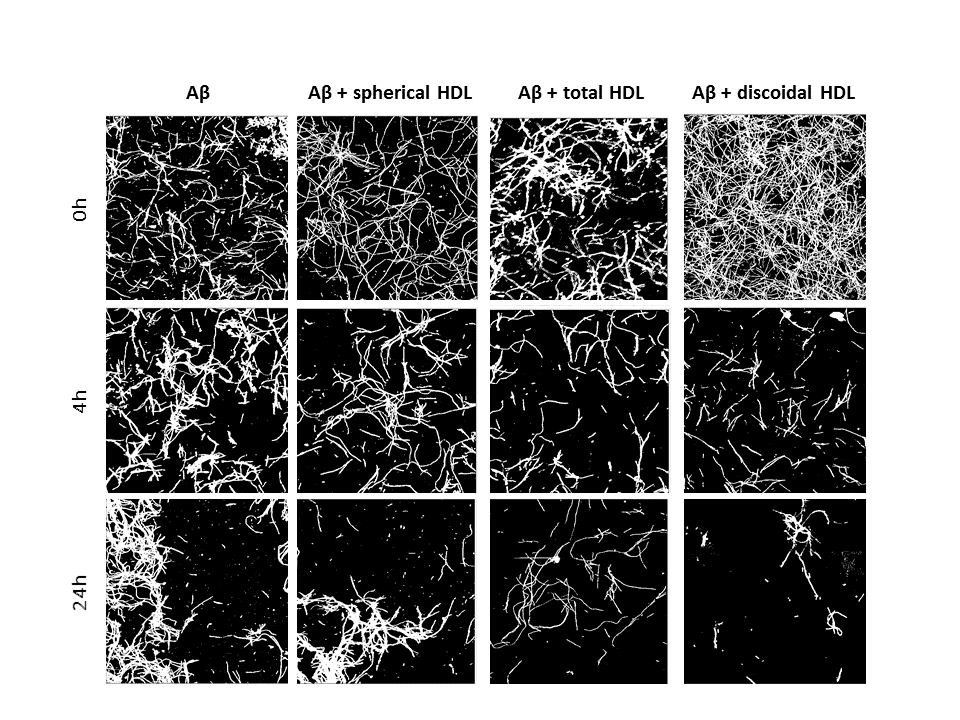

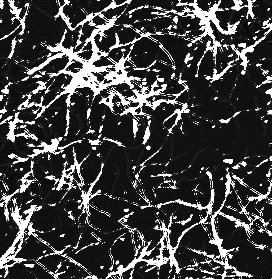


**Supplementary Figure 1.** A fixed height threshold (1.5 nm) is applied to the representative AFM images reported in the main text (Fig. 7A). This process allows quantification of the percentage of white pixels (pixel above a certain threshold), which is correlated to the fibrils crowding (total length and number).

In Supplementary Fig. 2A, the histogram of the height distribution of a representative AFM image at a certain step of the aggregation process is shown. Two peaks are distinguishable: the first higher peak centered on h = 0 nm is due to the statistical height distribution of the mica on which the fibrils are deposited, and the other lower peak centered around h = 3-4 nm is due to the presence of fibrils on the mica. Given the partial superposition of the two peaks, the choice of a height threshold could be somewhat arbitrary. Therefore, the consequences of a particular threshold selection on the time evolution of white pixels (i.e. pixels above that threshold) for the “Aβ + Discoidal HDL” column of Supplementary Fig. 1 was examined. The choice of a threshold between 0.5 nm and 2 nm is basically irrelevant as the white pixel percentage is independent of the threshold choice (Supplementary Fig. 2B-C).

| A)  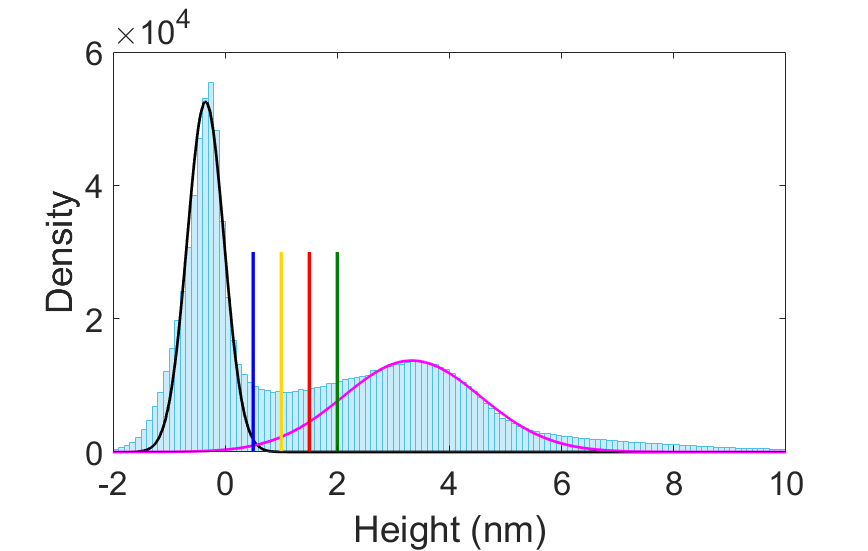 | |
| --- | --- |
| B)  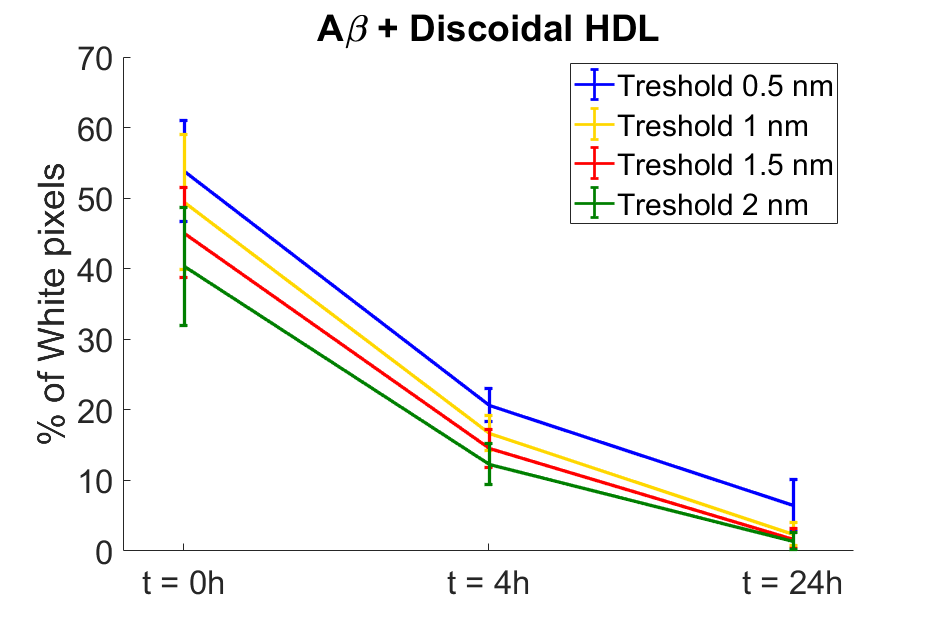 | C)  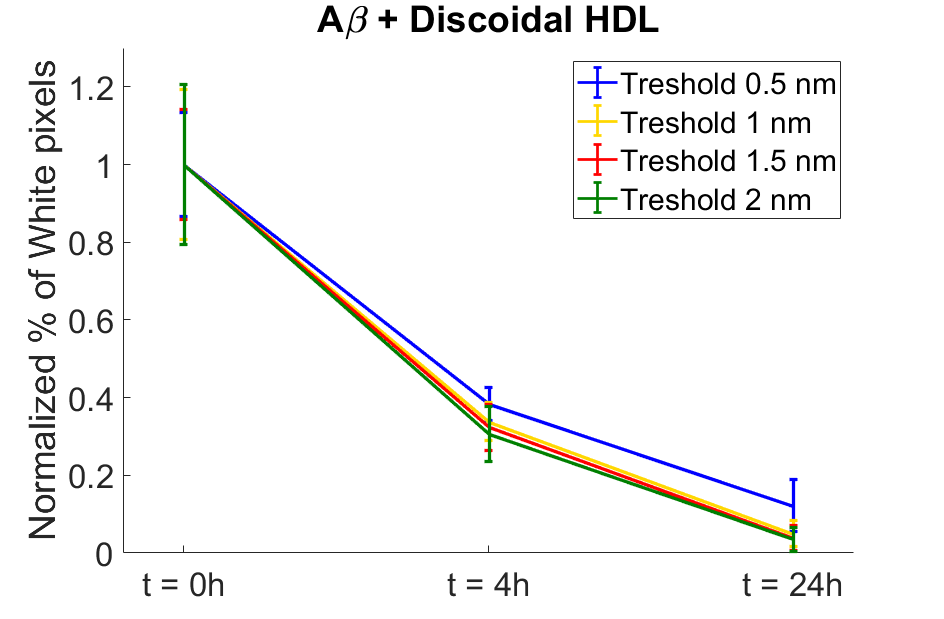 |

**Supplementary Figure 2**. (A) Representative height histogram of an AFM image of fibrils. Two peaks are visible, one for the mica surface (black Gaussian fit), and the other corresponding to the fibrils (magenta Gaussian fit). Four possible height threshold values are indicated: 0.5 nm (blue), 1 nm (yellow), 1.5 nm (red), 2 nm (green). (B) Percentage of pixels above a fixed height threshold (white pixels) as a function of the incubation time at 37°C evaluated for 4 different thresholds (0.5 nm in blue, 1 nm in yellow, 1.5 nm in red, 2 nm in green) for Aβ incubated with discoidal HDL. (C) Normalized percentage of white pixels above a fixed height threshold as a function of the incubation time at 37°C evaluated for 4 different thresholds (0.5 nm in blue, 1 nm in yellow, 1.5 nm in red, 2 nm in green) for Aβ incubated with discoidal HDL. The normalization is calculated with respect to the t=0h sample.

**1.3 Interaction of discoidal HDL with Aβ by computational modelling**

**
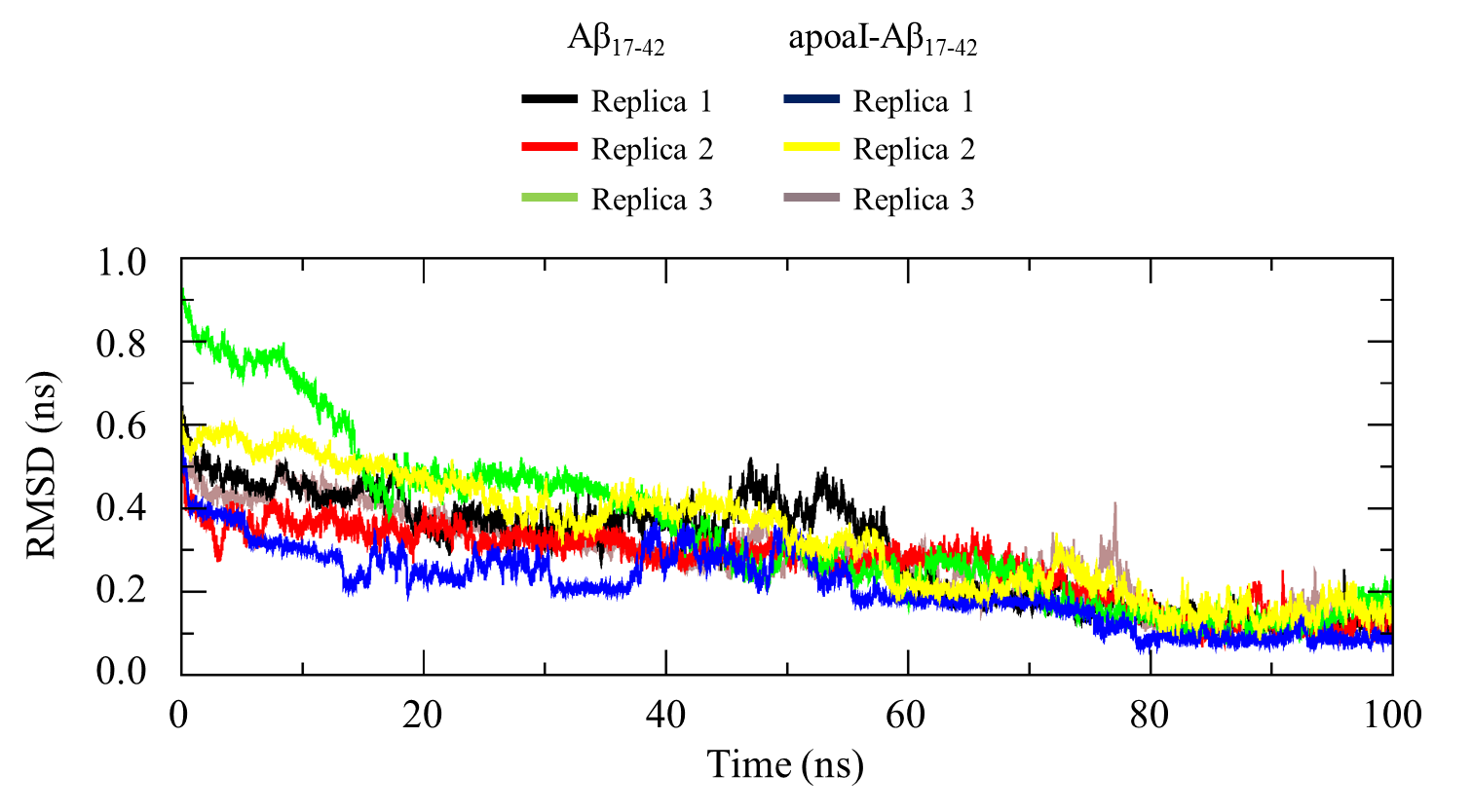
**

**Supplementary Figure 3. Protein structural stability by MD.** C-alpha/C-alpha Root Mean Square Deviation (RMSD) plot computed for each replica of the Aβ_17-42_ and apoA-I-Aβ_17-42_ molecular systems. Protein conformational stability was reasonably reached in the last 20 ns of the simulations.


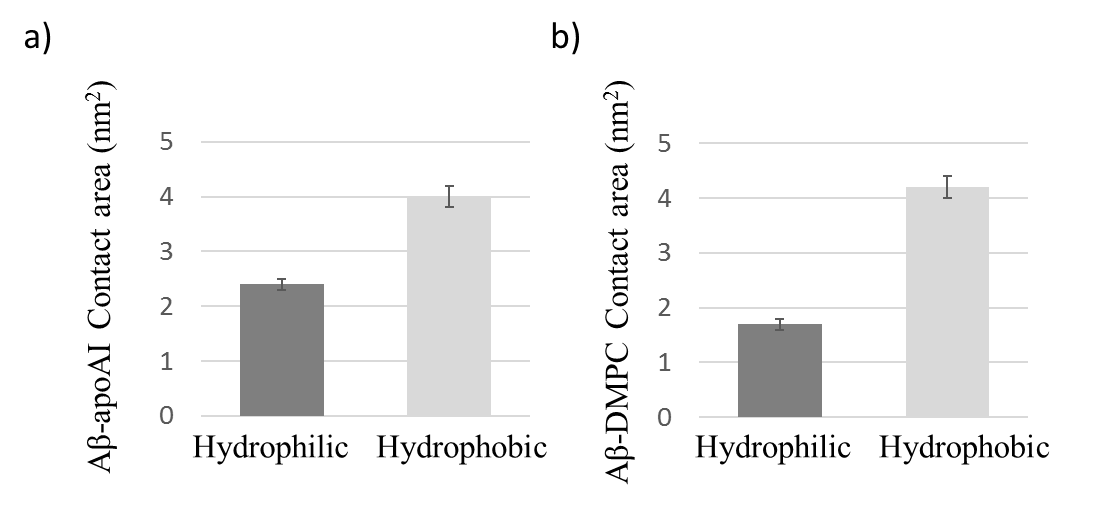


**Supplementary Figure 4. Interaction between Aβ_17-42_ and apoA-I evaluated by MD.** Histogram of the apoAI-Aβ_17-42_ contact surface characterized by a) protein-protein, i.e. Aβ-apoAI, and b) protein-lipid, i.e. Aβ-DMPC contact surface. In both cases, the hydrophobic contribution plays a major role in driving the apoAI-Aβ_17-42_ interaction.

**Supplementary Figure 5. Plot of Aβ_42_ efflux vs time (pmole/min) in the presence of Aβ alone (Ab), lipid-free apoA-I (lipid free), lipid-poor apoA-I (lipid poor) and discoidal apoA-I-HDL (disco) in the apical compartment of the transwell model.**

| hCMEC/D3 monolayers | |
| --- | --- |
| Treatment | TEER±SD (Ωcm^2^) |
| Untreated | 112±2.23 |
| Spherical apoAI-HDL | 115±3.01 |
| apoAI-HDL plasma pool | 114±3.89 |
| Discoidal apoAI-HDL | 113±2.17 |
| Lipid-free apoAI | 114±2.78 |

**Supplementary Table 1. TEER values of hCMEC/D3 monolayer after 3h of incubation with different HDL subclasses.**
